# How field of view impacts visual search strategies

**DOI:** 10.1167/jov.26.9.2

**Published:** 2026-09-01

**Authors:** Kaitlin Moat, Philip Grove, Stefanie Becker, Guy Wallis

**Affiliations:** 1 School of Psychology, University of Queensland, Brisbane, Australia; 2 School of Human Movement & Nutrition Sciences, University of Queensland, Brisbane, Australia

**Keywords:** virtual reality, attention, visual search

## Abstract

When searching for objects, our sensory resources are limited. According to the *zoom lens model* of attention, the visual system can flexibly adapt the size of the attentional spotlight applied to an array of items depending on task difficulty. When search tasks are relatively straightforward, attention can be distributed widely across space to process several items simultaneously. However, when discrimination becomes more difficult, the attentional spotlight constricts to concentrate resources to a limited area resulting in the processing of only one or very few items simultaneously at any one time. The present study aimed to empirically test the zoom lens account by assessing search performance while systematically altering the visible area in the search display (field of view [FOV]). We presented two search tasks of contrasting difficulty in a virtual reality environment with a head-contingent display. When the FOV became smaller, there were no differences in search times between the two search tasks, a result which seems to contradict the predictions of the zoom lens model. The zoom lens model predicts that restricted viewing in a demanding search task should exact a smaller cost because of the presence of the smaller attentional window. However, we did not find any evidence of such an effect. Results from an analysis of head and eye movements also suggested that when using both fixations and head movements to scan their environment, participants used head movements to reveal new areas of the visual scene in a manner largely disconnected from classic eye movement and attentional processes. Once the head came to rest, attentional shifts and eye movements were used to search within the newly revealed area.

## Models of visual search

Traditional attention models liken attention to a spotlight, focusing on relevant aspects of the visual field for deeper processing while ignoring irrelevant items (Posner, 1980). Initially thought to be of a fixed size, Eriksen and St James (1986) suggested that the attention spotlight instead functions like a zoom lens, adjusting based on search demands. In easy or parallel visual search tasks, such as finding a red target among green items, attentional resources can be distributed broadly with minimal attentional resources devoted to individual items (Eriksen & St James, 1986; Treisman & Gelade, 1980). In contrast, difficult tasks or serial searches, like locating a red square among green squares and red circles, require concentrated resources over a smaller area, resulting in a narrower zoom lens.

The zoom lens model posits that items within the spotlight can be processed simultaneously (in parallel) via a greater concentration of resources, with fewer resources available outside the spotlight. When the spotlight does not cover all stimuli, it must be shifted serially to process them. In easy feature searches, where the target differs by a single salient characteristic, detection is efficient even with a broad focus (Treisman & Gelade, 1980). A large attentional spotlight allows for simultaneous processing, resulting in faster search times. However, in serial searches the spotlight must narrow, concentrating resources across a small area and limiting the useful field of view (Eriksen & St. James, 1986). This smaller attentional window reduces the impact of irrelevant distractors (Belopolsky, Zwaan, Theeuwes, & Kramer, 2007), but it also means fewer items can be processed simultaneously, necessitating more attentional shifts and resulting in longer search times.

Evidence for the zoom lens model of attention is found in a variety of different visual search paradigms. For example, when participants were presented with an array of words and asked to complete either an easy search task (identifying a word or a person's name) or a difficult search task (identifying whether the middle letter of a string belonged to a specific letter set), participants were quicker to respond with a button press to a probe stimulus occurring in the middle of the visual field when the task was hard (LaBerge, 1983). There was no effect of location when the task was easy, suggesting that when the task was easy, attention was distributed widely across the whole array. This study prompted LaBerge to propose that attention could be concentrated on areas variable in size and is taken as early evidence toward the zoom lens models conception (Eriksen & St James, 1986).

Shen, Reingold and Pomplun (2000) tracked participants’ eye movements during visual search to assess the useful field of view in easy and hard search tasks. They asked participants to conduct a visual search in which task difficulty was manipulated by increasing the number of same-colour distractors. When the search became more difficult, more fixations were made, indicating that participants were more likely to serially check search items than when the task was easier. Similarly, Williams and Reingold (2001) also conducted a visual search task, where they varied the search items by three features: shape, orientation, and color. When the search task was easier, the target shared only one similar feature with the distractors, and in the more difficult task, the target shared two. Participants made more fixations when the search task was more difficult, indicating that the number of items processed during a single (attentional) fixation was reduced in the harder search tasks.

The attentional window surrounding a fixation has also been referred to as a functional visual field (FVF; Sanders & Houtmans, 1985). These functional visual fields are thought to have three distinct purposes or mechanisms (Wu & Wolfe, 2022). The resolution FVF defines the visual area around fixation in which a target can be identified when attention is also deployed at this location. The attentional FVF describes the area around a given fixation in which stimuli could be covertly attended to. Finally, the exploratory FVF describes the new location to which the eyes will move to if the target is not found in the attentional FVF. In easy visual search tasks, these exploratory eye movements are not necessary, as target stimuli are typically easy to locate within the attentional FVF, leading to fewer eye movements in an easy search task. Collectively, the evidence suggests that when a search task is easy, attention is usually distributed widely, allowing processing of a larger number of items during a fixation, resulting in fewer eye movements and fixations, and faster search times. When a visual search is hard, attentional resources must be pooled over a smaller area, resulting in the processing of fewer items during a fixation, resulting in more eye movements and fixations, and longer search times. To investigate this more systematically, previous studies have often used gaze-contingent search tasks.

Peripheral vision is also thought to be a key driver of where these attentional resources are directed (Vlaskamp & Hooge, 2006). When participants conduct a visual search task, it has been previously observed that the periphery assists in deciding where to look next. What has been less explored is how attention is directed when access to the periphery is limited.

## Gaze-contingent visual search tasks

Gaze-contingent search tasks limit the viewable area around fixation while a person is searching, which artificially constricts the FOV. In these tasks, the amount of viewable area of the visual field is contingent on where the gaze is fixated, with peripheral information around this fixation either masked or obstructed. Assessing eye tracking parameters (e.g., the number of fixations) at different FOVs enables investigation into how task difficulty interacts with the size of the effective attentional window to modulate search performance, allowing new insights into how task difficulty and the size of the useful FOV interact to affect search performance.

If the zoom lens model of attention is a correct descriptor of search behavior, we would expect to see differences in search performance with a restricted FOV across task difficulty. The zoom lens theory would predict that limiting the FOV should primarily impair performance in easy search tasks when the natural attentional window would be wide, and not in a harder search, when the attentional window is already limited to a few items. As attention can be distributed widely when a search task is easy, introducing a constricted FOV may inhibit performance, as it constricts this large attentional window. When a search task is hard, the attentional window is thought to be smaller. This means that artificially limiting the FOV may not necessarily result in worse search performance because the attentional window is already small. Hence, limiting the viewable area around fixation is one way to explore how limiting the useful FOV interacts with task demands.

Previous gaze-contingent studies artificially constrained the FOV around the focal area (Becker, Horstmann, & Remington, 2011). When participants completed a visual search task, the researchers found that with a more limited gaze-contingent window, participants took longer to find the target object, and made more fixations (Geisler, Perry, & Najemnik, 2006). Building on these earlier reports, Young and Hullman (2013) also manipulated the size of the gaze-contingent window. Overall, search times were longer when the gaze-contingent window was more constricted. However, analysing the data separately for easy and hard search revealed that search performance only declined sharply under restricted view conditions compared to a free viewing condition when the search task was easy. This work provides further support for the zoom-lens theory of attention, as search performance suffered from a limited, gaze-contingent FOV in the easy task, which could have been completed faster with a larger attentional spotlight.

Little work has been done to explicitly test the zoom lens theory of attention by constraining the FOV across different levels of task difficulty. Previous work in the visual search literature strongly suggests that there are differences in search patterns when the task is more difficult, which may be due to the need to limit the size of the attentional window. However, only a handful of studies have directly tested this prediction by artificially restricting the FOV using a gaze-contingent search window during easy and hard search tasks (Geisler et al., 2006; Young & Hulleman, 2013). Many previous gaze-contingent search studies kept the search difficulty constant, which does not allow for tests of the zoom lens theory as a function of task difficulty (David, Beitner, & Võ, 2021). Previous experiments also often required participants to keep their head stationary (Young & Hullman, 2013), which fails to consider the more natural search behavior in which we are free to move both our head and our eyes. To the best of our knowledge, the zoom lens theory of attention has not been tested outside of the laboratory in more ecologically valid conditions in which head movements are permitted.

## Head movements

The vast majority of research conducted on visual search has been carried out with head movements discounted or actively constrained (for example, Saurels, Peluso, & Taubert, 2024). Although this may be appropriate under strict laboratory conditions, it does not reflect normal everyday behaviour when searching for items or during navigation. In real life situations, we use both our eyes and our head movements to locate objects in our environment. In search tasks where participants are allowed to move their heads, we observe that eyes movements and head movements play different navigational roles when searching for objects in the environment (Solman, Foulsham, & Kingstone, 2017).

In one experiment, participants viewed scenes through a grey mask that was yoked to either their eye or their head movement (Solman et al., 2017). This mask contained a cutout window through which participants could view a natural scene. When the location of the window was controlled by eye movements (a gaze-contingent task), participants favored exploring information already available within the window. However, when the location of the window was controlled by head movements (a head-contingent task), participants used their head to make exploratory movements, revealing new areas of the scene. These new areas were then examined in further detail using eye movements. This suggests that in environments where head movements are necessary for navigation, these head movements may act as the exploratory FVF and that eye movements are favored for prioritizing and focusing on objects that are already in the available visual field. Importantly, participants with a smaller mask in the head-contingent condition made more head movements, suggesting they compensated for reduced visual information.

Thinking about these findings in relation to the zoom lens theory of attention raises some interesting questions. Moving the head during a search may not only expose new parts of the visual scene, but it could also help shift or resize the attentional window in space. If that is indeed the case, we may expect head-contingent search paradigms to show similar effects to gaze-contingent ones, where narrowing the amount of available information around a movement would result in a decrease in performance in easy search tasks but not in hard search tasks. However, if head movements serve a distinct navigational function rather than acting as an extension of eye movements, we may expect head movement patterns to differ from fixations. More specifically, we may expect head movements to take the role of the exploratory FVF, as participants may use their head to reveal new areas of their environment, and use their eyes to search the newly revealed area in more detail. This would perhaps result in head movements being less sensitive to task difficulty than fixations, such that the difference in head movement patterns between easy and hard search tasks would be smaller than the equivalent difference observed in fixation patterns.

One way to test these predictions is by using virtual reality (VR). VR provides a unique opportunity to conduct a visual search task where it is possible for participants to move both their head and eyes freely, and for experimenters to measure these movements simultaneously. A second motivation for using VR is that it is currently being used as a training tool in a variety of industries (Xie et al., 2021), as well as in attentional research (Stein, Watson, Lappe, Westendorf, & Durant, 2024). However; virtual reality headsets typically have a limited FOV compared to real life viewing conditions. Although modern headsets have a reported FOVs of up to 120 degrees (Vive, 2022), this is still considerably smaller than the average human visual field of approximately 180 degrees (Parsaee et al., 2021).

When performing a task in an environment with a limited FOV, it is perhaps unsurprising to discover that performance is impacted relative to unconstrained viewing. For example, in a pilot training task using a flight simulator where participants could move their heads to navigate (Karar & Ghosh, 2014), pilots made significantly more errors and missed more information over time with limited peripheral vision than with a wider FOV. Similarly, David, Beitner and Võ (2021) conducted a search task in virtual reality, where participants had to locate an object in a virtual room. In one condition, the peripheral information around a fixation was masked. On average, participants took longer to find targets and made more head movements compared to those with full vision. However, one limitation of these experiments in relation to our research interests is that task difficulty was not manipulated.

We chose to test the predictions of the zoom lens model when head movements can also be used to navigate a search display. To do this, we investigated search performance across different levels of task difficulty as well as for different FOV sizes. More specifically, we expected to replicate previous gaze-based experiments, where search times are significantly impacted by a small FOV in easy search tasks, but not in hard search tasks. However, we may also expect to see search times in the hard task to also be significantly affected by a constrained FOV.

The relationship between head and eye movements in limited-FOV environments raises some interesting questions. First, is the zoom lens theory of attention still an appropriate model of search behaviour when participants can use head movements, as well as eye movements, to navigate a display? Second, how would search behavior differ when participants are in an environment with a reduced FOV?

## The current task

The current experiment aims to test predictions of the zoom lens model under different FOVs. To do this, participants were assigned one of three levels of FOV: a small FOV, a medium FOV or an unconstrained FOV. The FOV was head-contingent, meaning that the FOV mask was yoked to the participants’ head movements. They then completed three blocks in their assigned constraint condition. Participants also completed one of two search task difficulties- either easy or hard.

Overall, search times in the hard search condition (where the distractors and the target are more similar) should be longer than in the easier search condition. We also hypothesize that task difficulty and FOV will interact, whereby search times will be more affected by a limited FOV when the task is easy than when the task is hard. According to the zoom lens model, we would expect to see less cost of a limited FOV when the task is hard, because participants would already be narrowing their attention to a localized area in space and, hence, not benefit as much from access to a wider FOV. Finally, we expect that conditions with a more limited FOV will generate more head movements.

As part of the overall research project, participants also completed a fourth and final block with the FOV widened to explore whether any additional head movements learnt during the first three blocks would persist, and whether the task difficulty would impact this. These results are beyond the scope of this article and will be reported elsewhere.

## Methods

### Participants

Sixty-six participants (44 female, 22 male) with ages from 18 to 55 (*M* = 23.9, *SD* = 6.8) completed four blocks of 45 trials of a virtual reality, head contingent visual search task. The mean ages and standard deviations for each condition are reported in Supplementary Appendix S1. Participants were recruited from the University of Queensland's undergraduate student pool and the paid participant pool. Participants recruited via the undergraduate student pool were compensated with course credit, and those recruited through the paid participant pool were given a $20 gift card for their time. Individuals with a history of motion sickness were excluded from this study. The task was conducted using the HTC Vive Pro Eye, using Unity 2019.4.3 and SRanipal Eye tracking software. The project was approved by the University of Queensland's ethics board (2023/HE001489) and is in line with the Declaration of Helsinki.

### Stimuli

**Figure 1.**
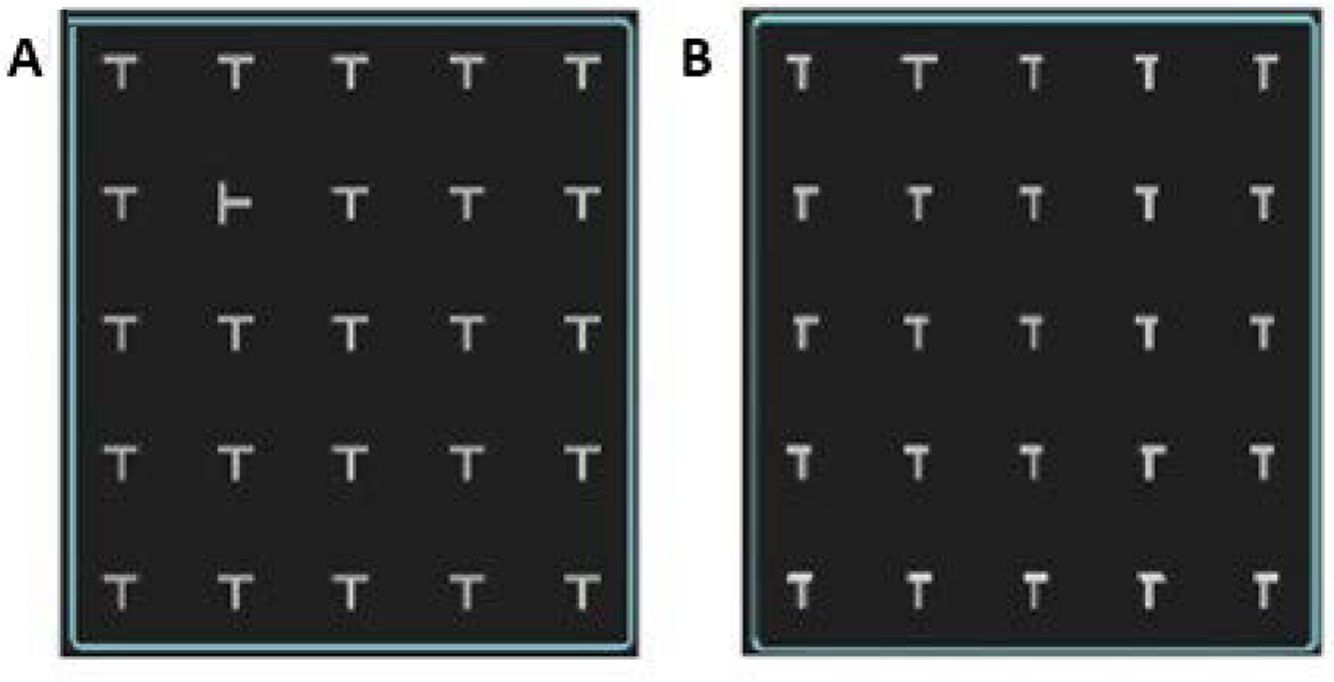
Examples of each search difficulty as shown in VR. An example of the search display in the easy search task is shown on the left, and the hard task on the right. Targets are located in the top left quadrant of both displays.

The search display contained a five-by-five array of white objects on a black background (Figure 1). Using Unity's scene editor, the stimuli were designed to be 12 cm wide and 12 cm tall and were located 1.9 m away from the participant. Use of these parameters created the illusion of stimuli being projected on a wall in front of the participant. To calculate the size of the stimuli in degrees of visual angle, measurements were taken from an experiment where reference stimuli were placed both in real life and in VR, in the same location (King & Wallis, 2026).

### Design

The experimental design was a two (Search Difficulty: Easy or Hard Search) by three (FOV Condition: unconstrained, a medium FOV, and a small FOV) between-subject design. Participants completed three experimental blocks of 45 trials, and there were eleven participants in each condition. For all three blocks of the experiment, participants completed the task with their assigned FOV condition.

**Figure 2.**
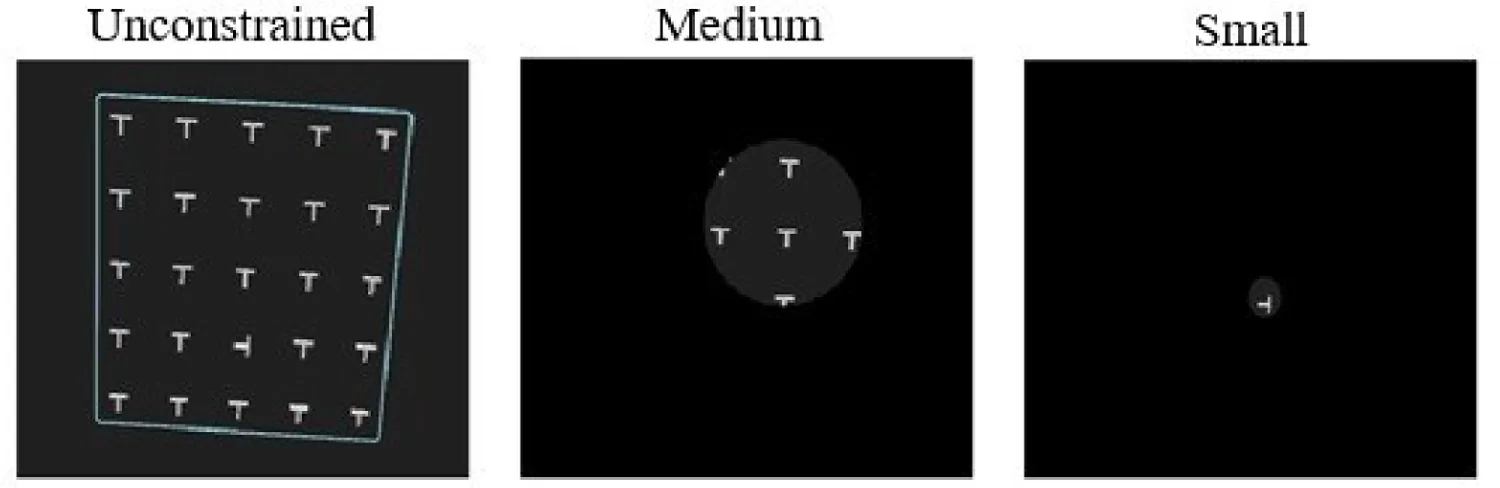
FOV conditions, as shown from within the VR headset, shown from the largest FOV on the left to the smallest on the right.

Participants either completed an easy search task or a hard search task. In the easy search task, the target was a 90° rotated T among upright T distractors (see Figure 1). In the hard search condition, the target was an upright, vertical T. Distractors were Ts with 30% of the top length removed on either the left or right side (Figure 1b). The stimuli in the hard search conditions were based on previous serial search paradigms (Peltier & Becker, 2017; Zhang & Houpt, 2020), but differed because they all had a uniform orientation. The decision to keep the orientation of distractors uniform and slightly change the presentation of the distractors was taken to reduce incidence of eye strain reported during pilot testing using the head mounted display. Participants were assigned to one of three FOV conditions: unconstrained FOV, medium FOV (32.12° wide) or a small FOV (8.02° wide; see Figure 2).

### Procedure

Before starting the experiment, participants completed five practice trials. Participants then completed three blocks of 45 trials in one FOV condition. The FOV constraint acted like a mask over the participants’ face, and participants had to use head movements to search the array. Once the participant had located the target, they had to fixate on the target for two seconds. This was done to eliminate any extraneous head movements that might have occurred by requiring participants to operate a keyboard. After this, there was a three second inter-stimulus interval. All data and materials are available at the projects OSF page (https://osf.io/ztg2k/).

## Results

Outliers were identified and removed at the level of individual trials and separately for each variable of interest when they were greater than two standard deviations from the mean. For search times, outliers were removed separately for the easy and hard search tasks. After the outliers were removed, averages for each participant were calculated for each of the three experimental blocks. Details on each outlier removal process for each variable of interest is included in each section of the results.

Search times were calculated by removing two seconds from the trial time, as the participant was required to fixate on the target for two seconds to proceed to the next trial. If the participant maintained fixation on the target for 95% of the time between their first fixation and the end of the trial, then the search time was calculated by taking the first fixation on the target from the time at the start of the trial. This avoids any inflated search times caused by the participant blinking during the two second fixation period after locating the target.

### Visual search

When analyzing search times across task difficulty, approximately 5% of the total trials were removed. In the easy search condition, 207 trials were removed, because of search times being 2 *SD* (3.52 seconds) greater than the mean (2.8 seconds). In the hard search condition, 287 trials were removed as they were greater than 2 *SD* (3.82 seconds) from the mean (3.81 seconds). A three (FOV: unconstrained, medium FOV and small FOV) by two (Search Difficulty: easy, hard) between-groups analysis of variance (ANOVA) was conducted to explore the effects of both search difficulty and FOV on search times, which were represented by each participant's average search time per block.

**Figure 3.**
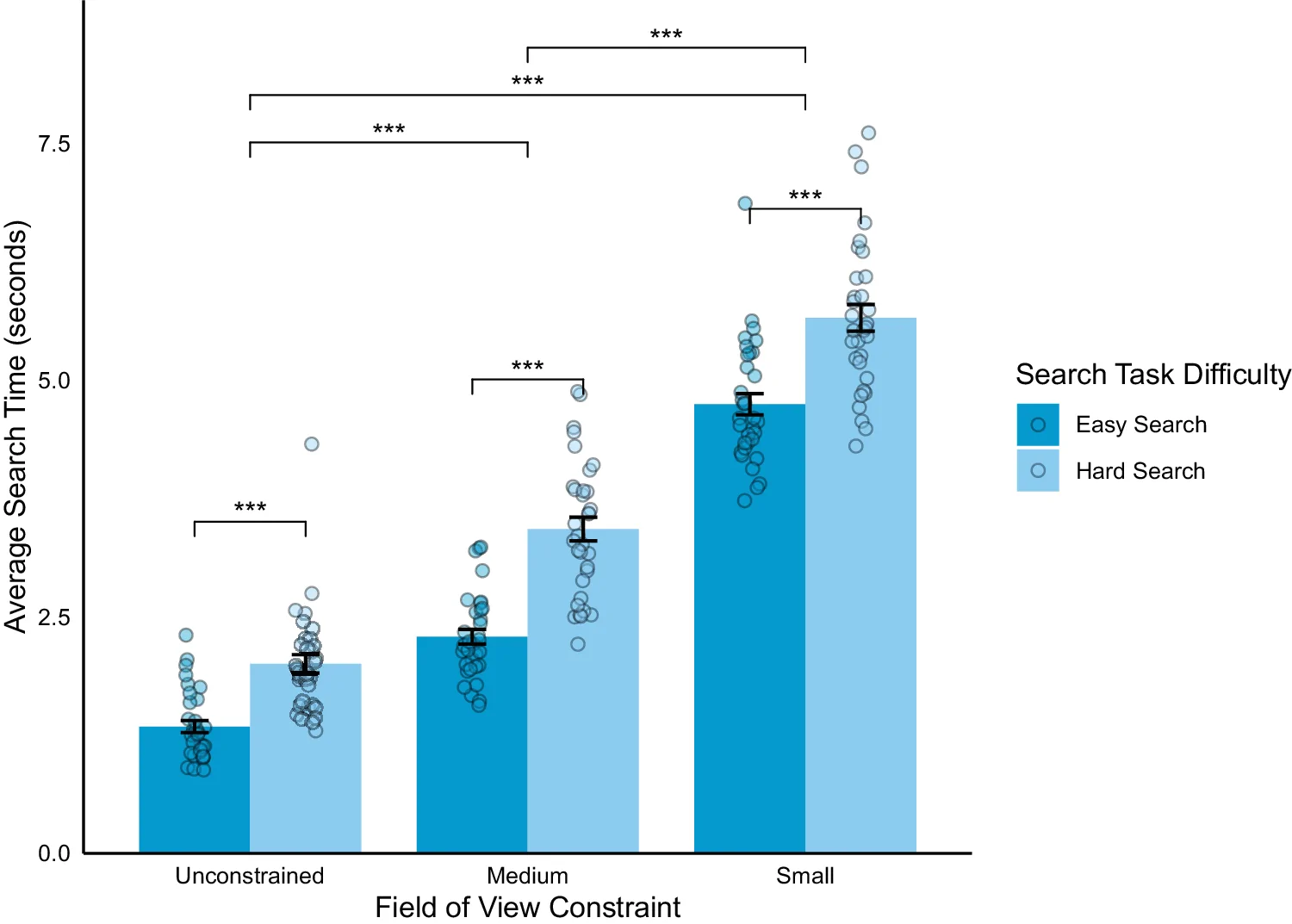
Average search time per FOV constraint, split by task difficulty. Error bars represent ±1 *SE*. Significance bars represent results of Tukey's HSD post-hoc comparisons.

A main effect of Search Difficulty was found (Figure 3), (*F*(1, 192) = 108.51, *p* < 0.001, $\eta_{p}^{2}$ = 0.36, *BF* > 100). Tukey's post hoc comparisons revealed search times in the easy task (average search time = 2.78 seconds) were significantly faster than the hard search (average search time = 3.69 seconds; 95% confidence interval [CI], 0.73–1.07; *p* < 0.001). Furthermore, a significant main effect of FOV constraint on reaction time was found, whereby conditions with a smaller FOV resulted in longer search times (Figure 3; *F*(2, 192) = 571.46; *p* < 0.001; $\eta_{p}^{2}$ = 0.85; *BF* > 100). Search times in the small FOV condition (average search time = 5.19) were significantly larger than both the medium FOV condition (average search time = 2.85; 95% CI, 2.09–2.59; *p* < 0.001) and the unconstrained condition (average search time = 1.67 seconds; 95% CI, 3.27–3.78; *p* < 0.001). Search times in the medium FOV were also longer than the small FOV (95% CI, 0.93–1.43; *p* < 0.001), suggesting that every time the FOV was reduced, the time it took to locate the target increased.

Recall that, according to the zoom lens model of attention, when a search task is more difficult, attention is focused on a smaller area of visual space, and when a search task is easy, attention can be distributed across a wider area. This means that when the FOV is artificially constricted, we would expect larger performance decrements when the search is easier, which should result in an interaction between search difficulty and FOV constraint. However, contrary to this prediction, the corresponding trends in the results failed to reach statistical significance (*F*(2, 192) = 2.49, *p* = 0.085, $\eta_{p}^{2}$ = 0.02, *BF* = 0.12).

### Eye movements

To assess search efficiency more directly, we also examined the eye tracking data. A fixation was categorized as a sample with a duration of 100 ms or longer where the Euclidian distance between samples and velocity did not exceed a threshold of 0.0416°/second, calculated by a change in fixation by 5° at a sampling rate of 120 Hz. This change in fixation value was chosen because it is half the distance between the stimuli, so this change in eye position would give the participant access to new, meaningful information about the task.

Removing trials with excessive fixations (more than 2 *SD* from the mean fixations per trial) resulted in a loss of approximately 5% of the total trials. In the easy condition, 231 trials were removed as the number of fixations were two standard deviations (7.6 fixations) greater than the mean (8.65 fixations). In the hard condition, 253 trials were removed because they were greater than 2 *SD* (8.85 fixations) from the mean (11.4 fixations). Fixations were then averaged across blocks for each participant for analysis. On average, there were approximately nine fixations per trial. For the following analysis, data from the first three experimental blocks were analyzed, because the FOV constraint was removed for all participants in the final block.

**Figure 4.**
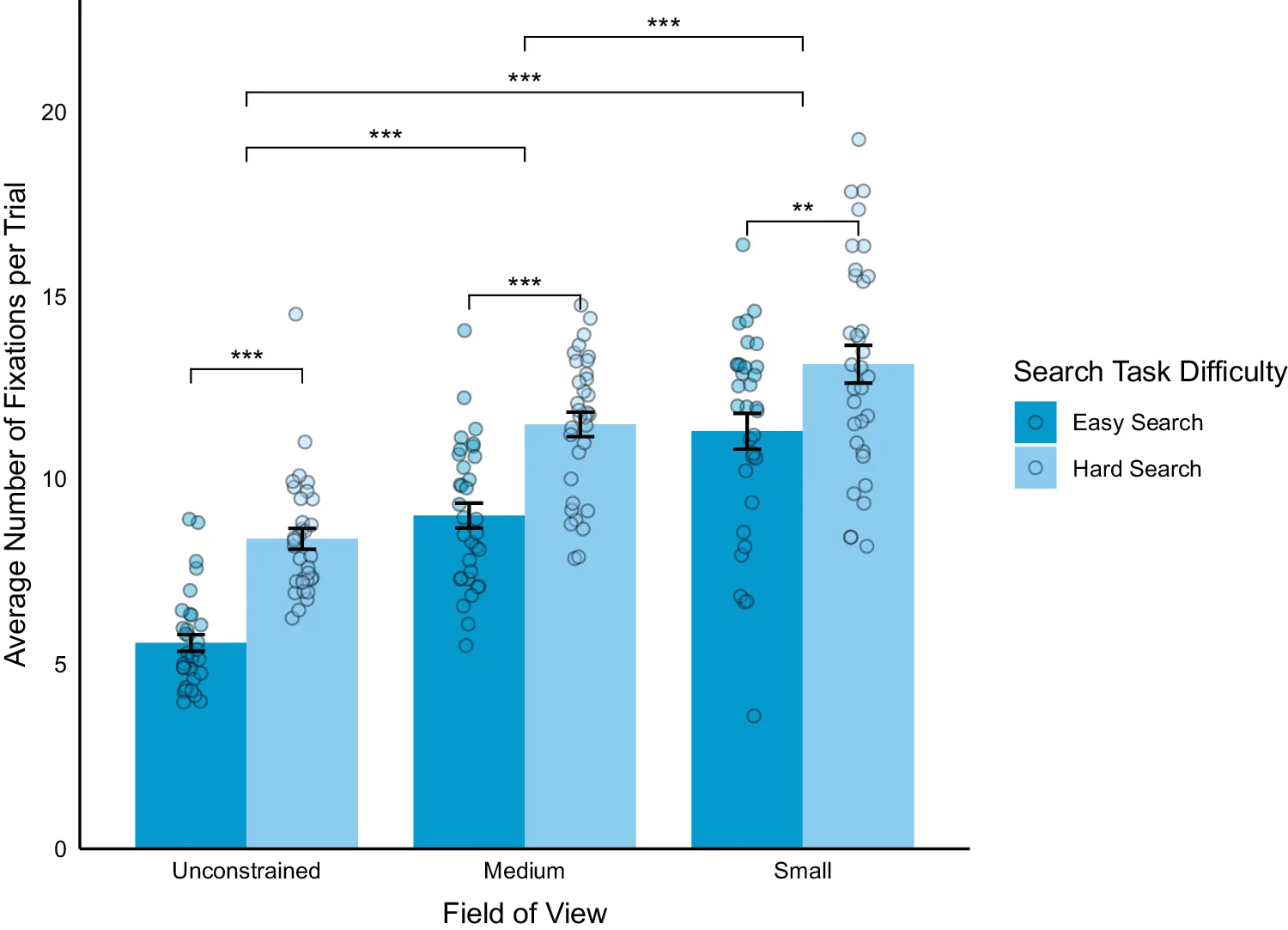
Average fixations across FOV constraint and task difficulty. Error bars represent ±1 SE. Significance bars represent results of Tukey's HSD post-hoc comparisons.

A two (Search Difficulty: easy, hard) by three (FOV: unconstrained, medium and small) ANOVA revealed a significant difference in the number of fixations across groups, whereby there were more fixations in the hard search task than the easy search task (*F*(1, 192) = 59.46, *p* > 0.001, $\eta_{p}^{2}$ = 0.24, *BF* > 100). There was also a significant effect of FOV constraint on the number of fixations, with smaller FOVs resulting in more fixations (*F*(2, 192) = 98.71, *p* < 0.001, *BF* < 100, $\eta_{p}^{2}$ = 0.50). There was no significant interaction (*F*(2, 192) = 0.916, *p* = 0.4, *BF* = 0.12, $\eta_{p}^{2}$ = 0.009). These results are illustrated in Figure 4.

When conducting a Tukey's HSD follow-up analysis, we found significant effects between FOV conditions. Overall, there were more fixations in conditions with smaller FOVs. The 95% CI from the Tukey HSD indicate the range of plausible values for the mean difference between groups. There were significantly more fixations in the small FOV condition (average number of fixations = 12.22) compared to both the medium FOV (average number of fixations = 10.26; 95%CI, −2.87 to −1.04; *p* < 0.001) and unconstrained conditions (average number of fixations = 6.77; 95% CI, −6.28 to −4.61; *p* < 0.001). Similarly, there were also more fixations in the medium FOV condition compared to the unconstrained condition (95% CI, 2.79–4.18; *p* < 0.001). The number of fixations per second is reported in Supplementary Appendix S2.

**Figure 5.**
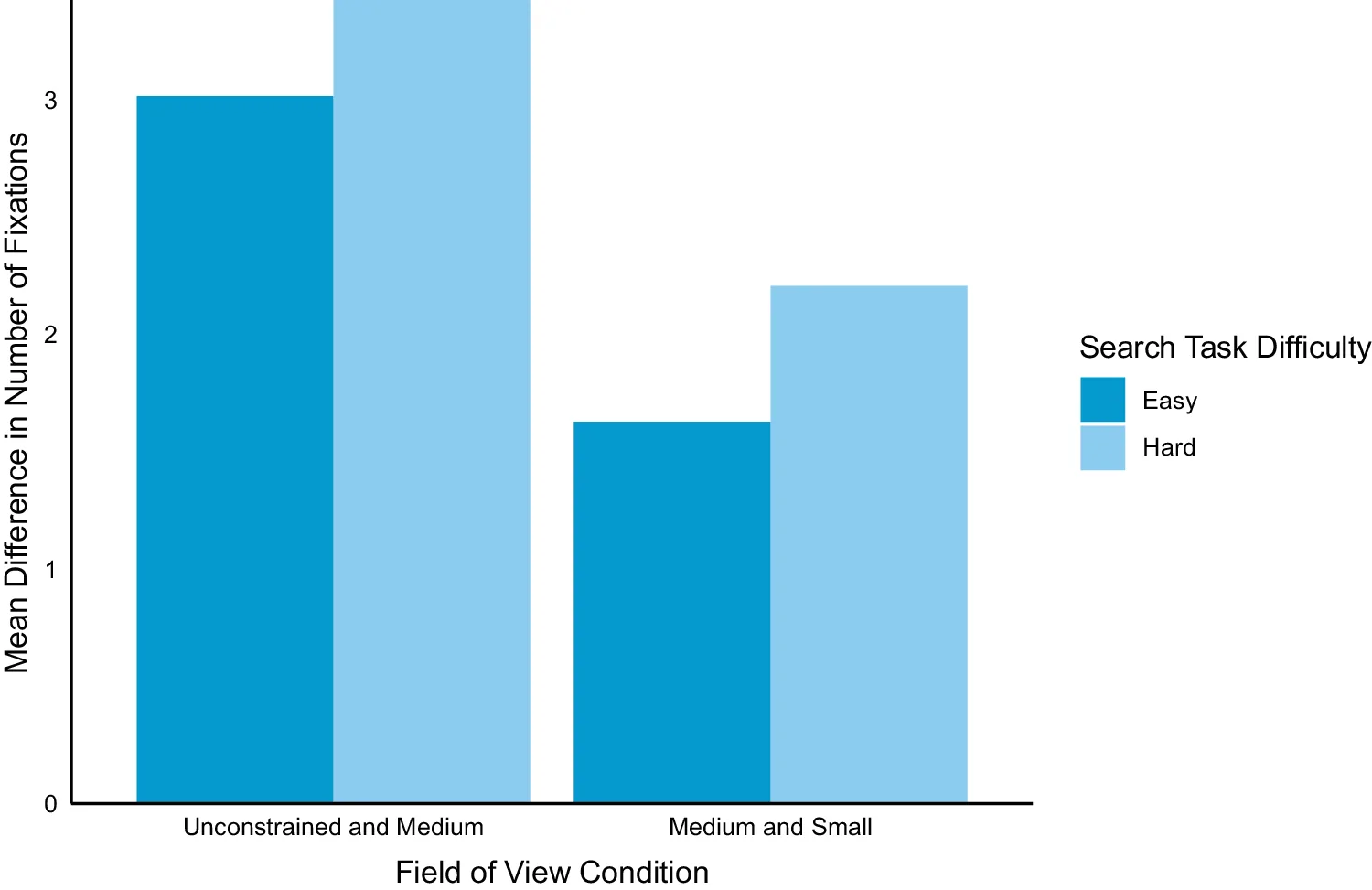
Difference scores of mean number of fixations across FOV conditions. X-axis values depict mean differences in fixation counts, derived by subtracting the medium FOV condition mean from the unconstrained FOV condition mean, and the small FOV condition mean from the medium FOV condition mean, respectively.

We also analyzed the mean differences between the number of fixations across FOV conditions to help visualise the direction of the pattern of the results. According to the zoom lens model, we would expect to see a larger cost of a limited FOV in the easy search task, opposed to the hard task. This may translate to larger mean differences in number of fixations in the easy task as the FOV increases, compared to the harder task. Contrary to this prediction, we did not observe a significant interaction, nor did the data trend in this expected direction. There was a slightly larger mean difference in average fixations when the FOV was reduced from medium to small in the harder task (mean difference = 2.2 fixations) than the easy task (mean difference = 1.63; Figure 5).

### Head movements

Overall, approximately 5% of the total trials (498 trials) were removed because they had an average number of head movements greater than 2 *SD* (*SD* = 2.6), above the mean (*M* = 3.3). The average number of head movements was then calculated for each participant. On average, there were approximately three head movements per trial.

**Figure 6.**
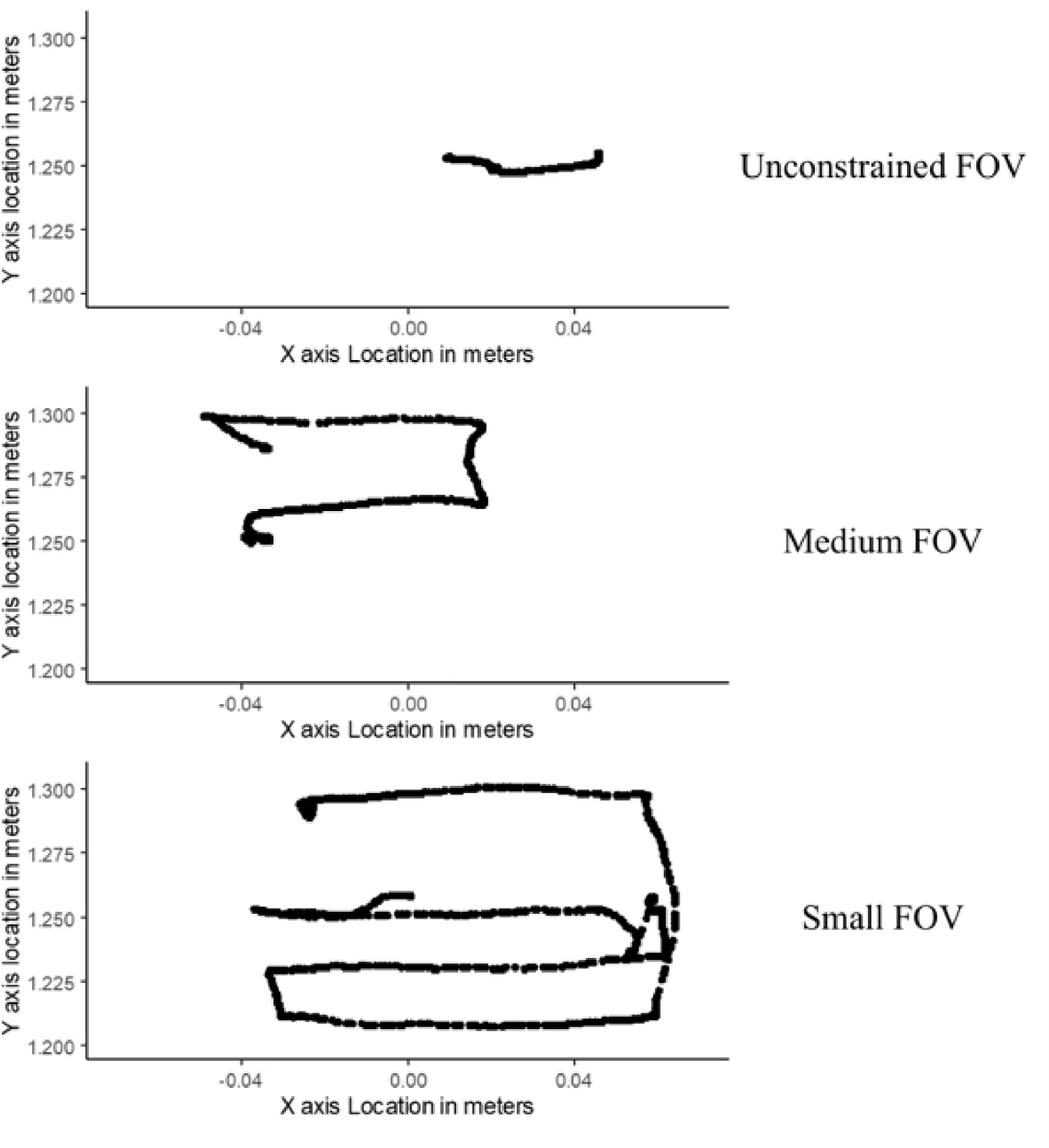
Head movements across a trial for participants in each FOV constraint. Trials were picked at random. A neutral head position is represented by an x axis location of 0, and a y axis location of 1.25, which is where the head is situated when a participant is seated, with the y-axis reflecting the participants average seated height. Positive x axis values represent a head movement to the right, negative x axis values represent a head movement to the left. Y axis values greater than 1.25 represent an upwards head movement, where y axis values lower than 1.25 show a downward head movement.

In this analysis, a head movement was categorized as any change in angular velocity that was larger than 5° per second (at a sampling rate of 120 Hz), to mirror that of the eye movement threshold. This value was chosen because it is half the distance between two stimuli, so a head movement this large would give the participant access to new, meaningful information about the task. Furthermore, if a head movement was continuous, but the direction of the movement changed meaningfully (from left to right, or from up to down), an additional head movement was recorded. An example of the head movements across trial can be seen in Figure 6 where each dot represents a participant's head location, as recorded by the VR headset.

**Figure 7.**
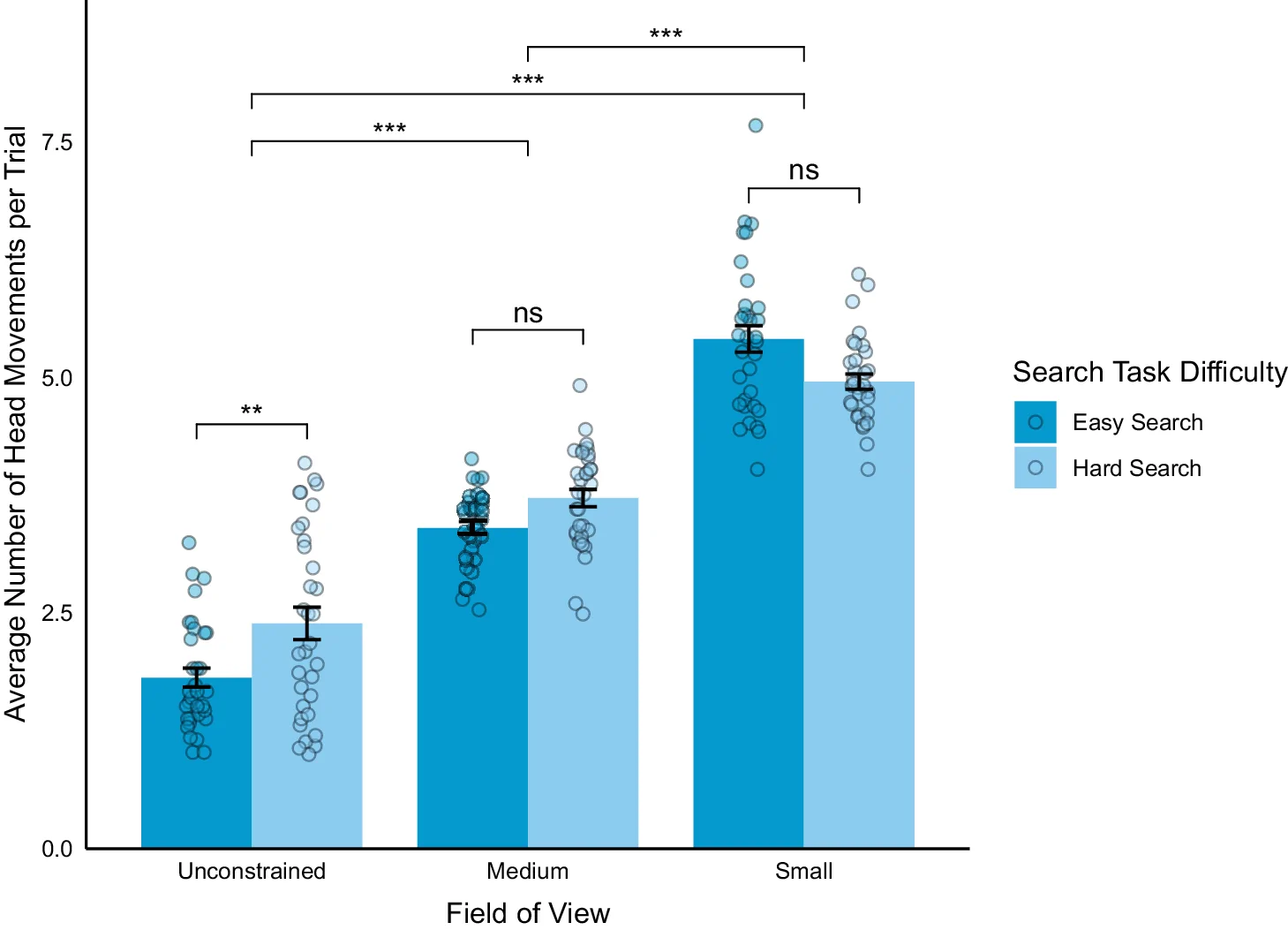
Number of head movements across FOV and task difficulty. Error bars represent ±1 SE. Significance bars represent results of Tukey's HSD post-hoc comparisons.

A two-way ANOVA was conducted to explore the effect of FOV and task difficulty on the number of head movements made, as a manipulation check to confirm that completing a task with a limited FOV resulted in more head movements (Figure 7). There was a significant effect of FOV on the number of head movements made, whereby the number of head movements increased as the FOV was reduced (*F*(3, 192) = 357.97, *p* < 0.001, $\eta_{p}^{2}$ = 0.79, *BF* > 100). There were significantly more head movements made when the FOV was small (average head movements = 5.19) than medium (average head movements = 3.57, 95% CI, 1.35–1.89; *p* < 0.001) and unconstrained (average head movements = 2.13; 95% CI, 2.80–3.35; *p* < 0.001).

There was no significant main effect of task difficulty on the number of head movements made. However, there was a significant interaction between the number of head movements made under different FOV conditions and task difficulty (Figure 7; *F*(2, 192 = 10.79, *p* < 0.001, $\eta_{p}^{2}$ = 0.10, *BF* = 0.58). Examining the effect of task difficulty within each FOV condition revealed the source of the interaction. Task difficulty only had a significant effect when the FOV was small, where participants made fewer head movements during the easy search task compared to the hard search task (95% CI, −1.04 to −1.06; *p* < 0.007). No significant effect of task difficulty was found in either the medium (95% CI, −0.78 to 0.15; *p* = 0.37) or unconstrained (95% CI, −0.02 to 0.92; *p* = 0.065) FOV conditions. This suggests the interaction is driven by more head movements during the easy task when the FOV was the smallest.

If the zoom lens model is a correct metaphor for explaining the deployment of attention, we may also expect to see a greater cost of a limited FOV on head movements for the easy task, compared to the hard task. This means we would expect to see a larger difference in the mean number of head movements as the FOV becomes smaller for the easy task compared to the hard task, as a limited FOV in this condition will preclude the dilation of the zoom lens across the display. To examine this, the difference scores between the means were calculated across FOV conditions for both the easy and hard conditions, and then plotted (Figure 7). When the FOV was smaller, we see a larger cost for the easy search task, opposed to the hard search task, as the mean difference in number of head movements is larger for the easy task (mean difference between a medium and small FOV for the easy task = 2 head movements, mean difference between a medium and small FOV for the hard task = 1.23 head movements). This implies that there is a larger cost for reducing the FOV when the task is easy, as participants have to make more head movements.

**Figure 8.**
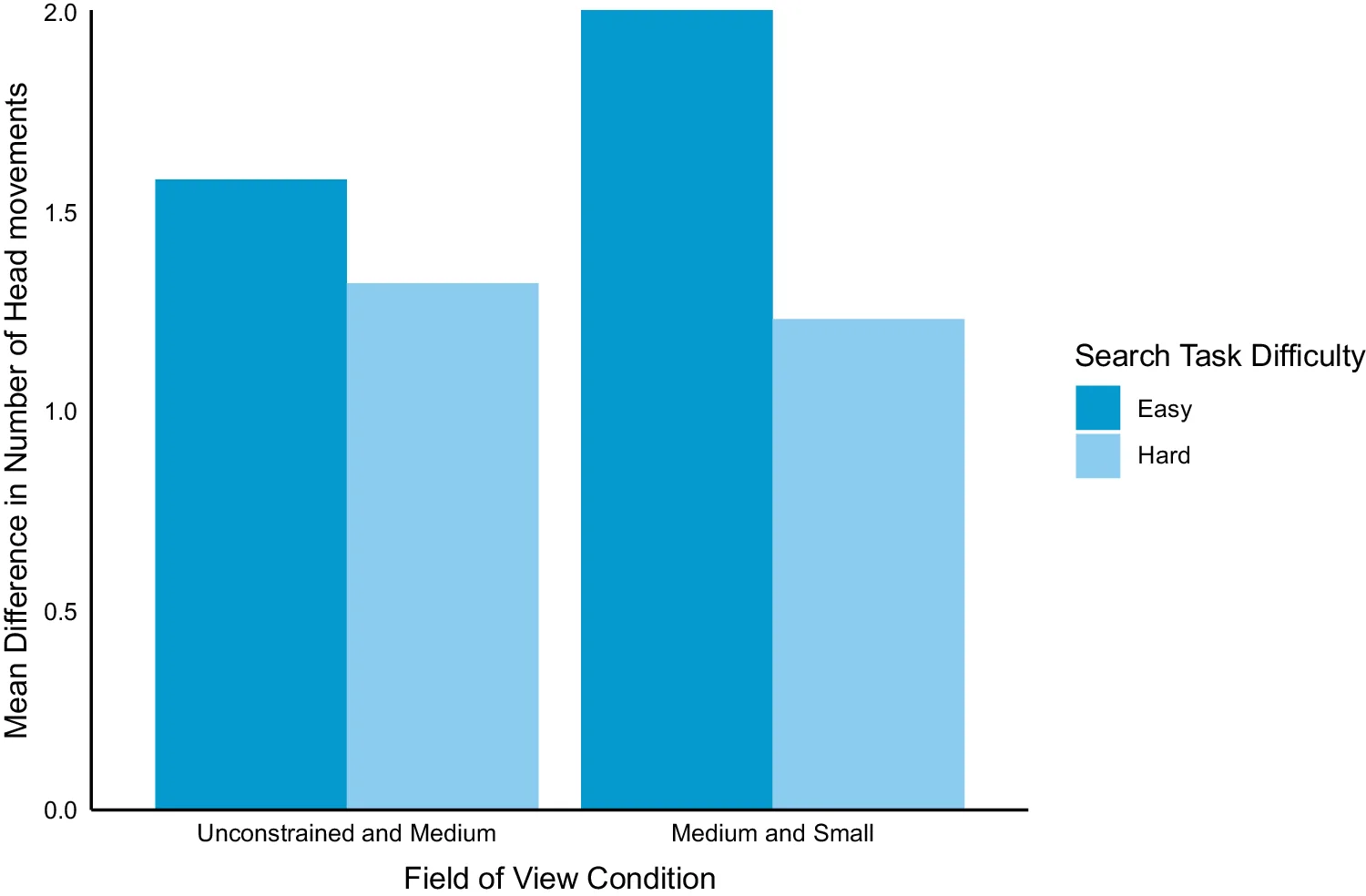
Difference scores of mean number of head movements across FOV conditions. X-axis values depict mean differences in fixation counts, derived by subtracting the medium FOV condition mean from the unconstrained FOV condition mean, and the small FOV condition mean from the medium FOV condition mean, respectively.

The pattern of results illustrates that the number of head movements increase as the FOV is reduced in the easy search task, yet remain relatively similar in the hard task. The mean difference between the medium and the unconstrained condition when the task is easy was 1.58 head movements, which increased to a mean difference of 2 head movements between a small and medium FOV. However, difference scores for the hard task remained similar as the FOV was reduced, because there was a mean difference of 1.32 head movements between the medium and no constraint compared to 1.23 head movements between the small and medium constraint. Figure 8 demonstrates these mean differences to help visualise the pattern of the results. An exploratory analysis was conducted to explore the number of fixations and head movements per second, which is provided in Supplementary Appendix S3.

## Discussion

Overall, our findings provide mixed support for the zoom lens model of attention. If this model is an appropriate metaphor for the deployment of attention in our task, then we would expect search times to be less affected by a constricted FOV when the task is hard, as the attentional window is already constrained. Despite this prediction, we were unable to detect a significant interaction between FOV and task difficulty.

When examining the visual search performance results, we found that as the FOV was reduced, the differences between search times in the hard search task were not significantly smaller than between search times in the easy search (as there was no significant interaction between search difficulty and FOV). This null effect is contrary to our prediction, as we expected there to be a larger decrement in search times with a smaller FOV when the task was easy. This finding is inconsistent with both the zoom lens model of attention, and previous findings that suggest the useful field of view shrinks for hard searches and widens for easier ones (Young & Hulleman, 2013). Despite this null result, our *p*-value for this interaction was approaching significance (*p* = 0.085). Future studies could aim to replicate this finding with larger samples.

Our inability to detect a significant difference in search times between task difficulty as the FOV became smaller may suggest that the loss of peripheral information is detrimental to search performance regardless of task difficulty. Indeed, it has been previously suggested that peripheral vision may be the key determinant of search performance, because it allows individuals to create a pooled visual representation of the search stimuli, which allows for differences in this representation to be spotted more easily (Rosenholtz, Huang, Raj, Balas, & Ilie, 2012). Perhaps this is one explanation for our pattern of results.

There were significantly more fixations when the task was hard, thus implying that the hard search was conducted in a more serialized manner than the easy search. In the unconstrained FOV condition, participants made the lowest number of fixations. Perhaps this could be because participants in the unconstrained FOV condition could covertly shift their attention to locate objects more quickly, allowing them to locate the target without moving their eyes.

Our results show that as the FOV got smaller, participants made more fixations. This result could also be explained by the fact that search times were longer in these conditions, therefore giving the participants more time to make fixations. A numerical inspection of mean differences (shown in Figure 5) does show that participants made more fixations as the FOV got smaller in the hard search compared to the easy search, although this interaction failed to reach significance.

When the FOV was smaller, participants made more head movements. This suggests participants needed to make a head movement to see a new area of the search display. This process then had to be repeated many times until the target was revealed. We also observed a significant interaction between FOV and task difficulty for head movements. When the search was easy and the FOV became smaller, participants made significantly more head movements, while the number of head movements made when the search was hard remained similar. This finding suggests that limiting the FOV for an easy search resulted in the need for more head movements.

We also observed dissociation between head and eye movements across task difficulties. When the FOV was small or medium, there was no effect of task difficulty on the number of head movements. This may be due to the nature of the task, as especially in the small FOV condition, participants had to make head movements to view all elements in the display. However, there was a significant difference in the number of fixations across task difficulty, where there were more fixations when the task was harder. This suggests that participants may have used their head to navigate the display but used their eye movements to actually search for objects within the viewable area. This finding echoes that of a previous study, which also found that in virtual environments, head movements were used largely for exploration, and eye movements were used to locate targets (Solman et al. 2017).

The difference between our hypothesised search time interaction and our observed results could also suggest a discrepancy in how we navigate a scene when we use both our eyes and head movements. Previously, visual search tasks have often been conducted with a strict control of head movements, with participants usually constrained via a chin rest (for example, Saurels et al., 2024). It could be the case that the inclusion of these head movements changes the nature of the search, rendering the zoom-lens model of attention less applicable. In our experiment, we found that as the FOV was reduced, the number of head movements and the number of eye movements increased. However, we see that FOV explains approximately 78% of the variance in the number of head movements, while it only explained 24% of the variance in the number of fixations made. This suggests that when the FOV was smaller, participants may have relied more on navigating the search array using their heads, rather than their eyes.

Our results suggest that head movements and eye movements could potentially be decoupled during navigation in this task. If participants did indeed use their heads to shift or resize their attentional window, we would expect to replicate the interaction in search times found in gaze contingent studies (Young & Hulleman, 2013). However, during our experiment, participants primarily used their head movements to reveal new areas of their visual field and then used their eye movements to search within this newly revealed area. This two stage process of using the head movements to reveal new areas of the environment and then using eye movements to search the newly revealed region would explain why task difficulty significantly influenced the number of fixations made, but not the number of head movements.

Our findings align with those found by Solman et al. (2017). Similarly, they also demonstrated that when a FOV constraint was yoked to head movements, participants used their head to reveal new areas of their environment but used their eyes to actually search the display. Our findings also suggest that similarly, in our paradigm where participants are using their heads to assist with navigation, head movements take the role of the exploratory FVF, whereas eye movements retain the role of the resolution and attentional FVF.

Taken together, it appears that introducing the ecological variable of head movements into search may interrupt behaviour predicted by the zoom lens model. Although we found significant interactions in the head movement data consistent with zoom lens predictions, we did not find the predicted interaction between search difficulty and FOV in search times. Overall, our findings suggest that head movements may serve a navigational function during search tasks that may be decoupled from attentional processes.

## Supplementary Material

Supplement 1

Supplement 2

Supplement 3
