# Supplementary material for: How field of view impacts visual search strategies
Source: J Vis. 2026 Sep 1;26(9):2. doi: 10.1167/jov.26.9.2 (PMC13549409; doi:10.1167/jov.26.9.2)
Supplement: Supplementary file 1 [file jovi-26-9-2_s001.docx]

**Appendix 1**

**Mean ages across FOV and task difficulty**

|  | Small FOV | Medium FOV | Unconstrained FOV | Average |
| --- | --- | --- | --- | --- |
| Easy Search | 22.4 (3.8) | 29.5 (10.5) | 23.55 (4.63) | 25.28 (7.5) |
| Hard Search | 24.6 (10) | 21.3 (2.6) | 21.6 (2.9) | 22.8 (6.2) |
| Average | 23.8 (7.4) | 25.4 (8.5) | 22.6 (3.9) |  |

*Note.* Standard deviations in brackets
