# Supplementary material for: How field of view impacts visual search strategies
Source: J Vis. 2026 Sep 1;26(9):2. doi: 10.1167/jov.26.9.2 (PMC13549409; doi:10.1167/jov.26.9.2)
Supplement: Supplementary file 2 [file jovi-26-9-2_s002.docx]

**Appendix 2**

An apparatus was measured which displayed buttons in VR, which were also located in the real world (King & Wallis, 2026). Measurements in VR are taken from the camera (front of the VR headset) and the locations of the apparatus in the Unity editor. Real life measurements were obtained by measuring from the nasion to the apparatus. Overall, we see a difference of approximately one degree between the two modalities. This could be due to observer movement between system measurements, or because the nasion is closer to the observer than the headsets’ location in Unity.

Overall, these measurements provide us with a validated measurement of visual angle between objects in VR environments.

All angles were calculated using the law of cosines.

**Degrees Calculations**

In VR, at a distance of 70.3cm, an object with a width of 18.7cm subtends 15.15 degrees of visual angle.

Therefore,

1° = $\frac{18.7}{15.15}$

1° = 1.23cm (at a viewing distance of 70.3cm).

This ratio can therefore be used to calculate the width of objects at various distances.

VR Measurements

Observer

61.8cm

67.4cm

70.3cm

70.3cm

67.9cm

63.4cm

18.2cm

17.6cm

18.7cm

17.4cm

18.5cm

15.5°

14.5°

15.3°

14.3°

15.7°

Real Life Measurements

Observer

64cm

65cm

63cm

63cm

65cm

64cm

18.5cm

18.5cm

18.5cm

18.5cm

18.5cm

16.6°

16.5°

16.8°

16.5°

16.6°
