# Supplementary material for: How field of view impacts visual search strategies
Source: J Vis. 2026 Sep 1;26(9):2. doi: 10.1167/jov.26.9.2 (PMC13549409; doi:10.1167/jov.26.9.2)
Supplement: Supplementary file 3 [file jovi-26-9-2_s003.docx]

**Appendix 3**

We conducted an additional analysis to explore the number of fixations (and the number of head movements) per second as suggested. We performed a three (FOV constraint: small, medium and unconstrained) by two (easy or hard) ANOVA on the number of fixations per second (Figure 1). We found a significant main effect of FOV constraint on number of fixations per second (F(1, 192) = 179.23, p < .001, η² = 0.65, BF > 100), where participants made significantly fewer fixations per second as the constraint increased.

We also found a significant main effect of search task difficulty (*F*(1, 192) = 5.17, *p* = 0.02, $\eta_{p}^{2}$ = 0.03, *BF* = 0.35), whereby participants made more fixations per second on average when the task was easy (3.55 fixations per second) than when it was hard (3.36 fixations per second). A Tukey’s follow up analysis revealed that this main effect was driven by the medium constraint condition, where participants made significantly more fixations in the easier version of the task (95%CI[-0.99, -0.15], *p* = 0.001). There were no significant differences in search difficulty when there was no constraint or a small constraint.

**Figure 1**

*Number of Fixations per Second across Different Task Difficulties and levels of FOV Constraint*

**

Repeating the same analysis for head movements revealed a similar pattern. We observed a large main effect of search difficulty (*F*(1, 192) = 32.7, *p* < .001, $\eta_{p}^{2}$ = 0.15, *BF* > 100), where there were significantly more head movements found when the task was easy (1.36 movements per second) compared to when it was hard (1.08 movements per second). Again, this main effect is driven entirely by a significant difference in the medium FOV condition (95%CI[-0.62, -0.13], *p* < 0.001). There were no significant differences in search difficulty when there was no constraint or a small constraint.

**Figure 2**

*Number of Head Movements per Second across Different Task Difficulties and levels of FOV Constraint*

***

The additional head and eye movements in the easy condition when there is a medium constraint reveals that the participants were likely using a strategic search pattern to locate objects in the display. When the target was easy to find, participants increased their likelihood of finding the stimuli by quickly scanning the display with their head and eye movements. This strategy appears to have worked, reflected in shorter trial times in the easy condition when the constraint was medium. However, participants could not use this strategy when the task was hard, as quickly scanning the display would not help them find the target any quicker.
